# Study on the Optimal Interval of Monitoring Following Gastric Polyp

**DOI:** 10.1155/DTE.6.59

**Published:** 2000

**Authors:** Minoru Kawaguchi, Toshihiko Saito

**Affiliations:** Fourth Department of Internal Medicine Tokyo Medical University 6-7-1 Nishishinjuku Shinjuku-ku Tokyo 160-0023 Japan

## Abstract

The present study was conducted to determine how long hyperplastic polyps (HPs) and fundic gland hyperplastic polyps (FGPs) should be endoscopically followed up. Our findings showed that in the case of FGPs, yearly endoscopy is not required and it is sufficient to repeat X-ray and compare films with those obtained in the previous year. In contrast, yearly follow-up by endoscopy is necessary in the case of HPs.


*Diagnostic and Therapeutic Endoscopy*, Vol. 6, pp. 59–66
Reprints available directly from the publisher
Photocopying permitted by license only

© 2000 OPA (Overseas Publishers Association) N.V.
Published by license under
the Harwood Academic Publishers imprint,
part of The Gordon and Breach Publishing Group.
Printed in Malaysia.

### Study on the Optimal Interval of Monitoring Following Gastric Polyp

MINORU KAWAGUCHI* and TOSHIHIKO SAITO

*Fourth Department of Internal Medicine, Tokyo Medical University, 6-7-1 Nishishinjuku, Shinjuku-ku, Tokyo 160-0023, Japan*

*(Received 21 June 1999; Revised 24 August 1999; In final form 21 September 1999)*

**The present study was conducted to determine how long hyperplastic polyps (HPs) and fundic gland hyperplastic polyps (FGPs) should be endoscopically followed up. Our findings showed that in the case of FGPs, yearly endoscopy is not required and it is sufficient to repeat X-ray and compare films with those obtained in the previous year. In contrast, yearly follow-up by endoscopy is necessary in the case of HPs.**

*Keywords:* Follow-up interval, Fundic gland polyp, *Helicobacter pylori*, Hyperplastic polyp

### 0. INTRODUCTION

Gastric polyps (benign epithelial elevated lesions in the stomach) are one of the diseases gastroenterologists most often encounter in daily clinical practice. They are also often discovered by X-ray in mass examination.

Discovery of gastric polyps in X-rays conducted to detect stomach cancer is followed by endoscopy for more detailed examinations. Even if polyps are found benign as a result of detailed examinations, it is recommended at present that patients receive follow-up examinations one year later. If lesions are found in X-rays the next year, detailed examinations by endoscopy is repeated, resulting in endoscopic procedures every year.

In order to determine if yearly endoscopic monitoring is necessary, we studied the fate of foveolar type hyperplastic polyps (HPs) and fundic gland hyperplastic polyps (FGPs), which account for a great majority of gastric polyps.

### 1. SUBJECTS

Included in the present study were the following patients who, undergoing endoscopy in Tokyo Medical University Hospital in 1994, were histologically diagnosed as having HPs or FGPs, and who were subsequently monitored for at least one year: (1) 95 HP patients (male : female ratio, 1.4 : 1; mean age, 61.3) and (2) 84 FGP patients (male : female ratio, 1 : 1.8; mean age, 50.4) (Table I).

* Corresponding author. Tel.: +81-3-3342-6111.

TABLE I Classification of subjects

| | HP | FGP |
|---|---|---|
| Subjects | | |
| No. of cases | 95 | 84 |
| Male : female ratio | 56 : 39 = 1.4 : 1 | 30 : 54 = 1 : 1.8 |
| Mean age | 61.3 | 50.4 |
| Size of polyps | | |
| < 5 mm | 25(5) | 67(10) |
| < 10 mm | 41(18) | 16(1) |
| < 15 mm | 39 | 1 |
| < 20 mm | 21 | 0 |
| > 20 mm | 11 | 0 |

( ): Morphological change.

TABLE II Morphological changes

| | HP | FGP |
|---|---|---|
| No change | 72 (75.8%) [28 M] | 73 (86.9%) [34 M] |
| Increased (size or number) | 16 (16.8%) [52 M] | 7 (8.3%) [50 M] |
| Reduction (size or number) | 7 (7.4%) [54 M] | 4 (4.8%) [48 M] |
| Total | 95 (100%) | 84 (100%) |

[ ]: Average time period of follow-up.

Because examinations were conducted by endoscopy, the size of polyps could not be accurately determined. Compared to the size of the cap of the forceps for biopsy, however, the size of most hyperplastic polyps was estimated to range between 5 and 10 mm, while some seemed to measure 20 mm or more. Most of the FGPs measured 5 mm or less. All FGPs except one which was subjected to polypectomy (and which measured 15 mm) measured 10 mm or less (Table I).

### 2. METHODS

Endoscopic films were retrospectively reviewed to determine changes in the morphology and number of polyps during monitoring, whether polypectomy was conducted, coexistence of dysplastic foci in polyps, and the presence or absence of tumorous lesions in other areas.

Biopsy specimens were retrospectively reviewed to determine the presence or absence of *Helicobacter pylori* (*H. pylori*) and the degree of inflammatory cell infiltration. The presence or absence of *H. pylori* was determined using the Hematoxylin–Eosin and Giemsa staining technique. Inflammatory cell infiltration was scored using the updated Sydney system [1], which evaluates inflammation-related items based on a 4-grade scale (none, 0; mild, 1; moderate, 2; severe, 3).

### 3. RESULTS

### 3.1. Morphological Changes

Existing polyps increased in size or the number of polyps increased in 16 of the 95 HP patients (16.8%), while existing polyps showed reductions in size or the number of polyps decreased (partly due to exfoliation) in 7 (7.4%). No morphological changes were seen in 72 patients (75.8%).

Existing polyps increased in size or the number of polyps increased in 7 of the 84 FGP patients (8.3%), while existing polyps showed reduction in size or the number of polyps decreased (partly due to exfoliation) in 4 (4.8%). No morphological changes were seen in 73 patients (86.9%) (Table II).

Most HPs with morphological changes measured 5–10 mm, while most FGPs with morphological changes measured 5 mm or less. All these polyps measured 10 mm, suggesting that the cause of morphological changes is not related to the sizes of polyps, but their nature.

These findings suggest that HPs are more liable to show morphological changes than FGPs.

### 3.2. Endoscopic Polypectomy

Endoscopic polypectomy was conducted with respect to 13 lesions in 10 of the 95 HP patients (10.5%). Dysplastic foci were observed in 4 of the 13 lesions (30.8%) (Figs. 1–3).

Endoscopic polypectomy was conducted with respect to only one lesion in 1 of the 84 FGP patients (1.2%). No dysplastic foci or cancerous foci were present in this lesion.

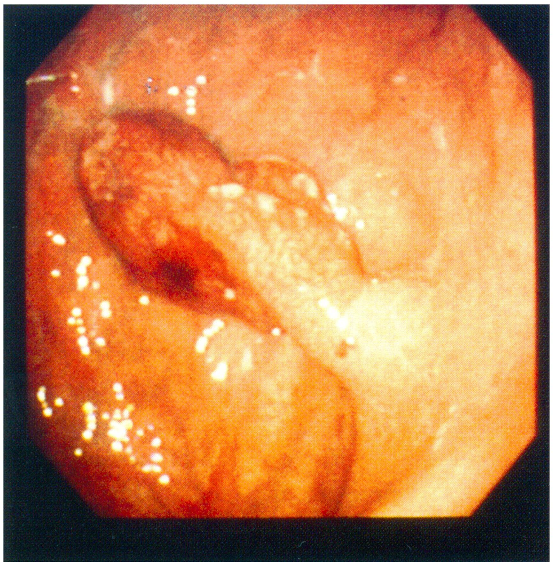

FIGURE 1 Case 1. 42-year-old man. A Yamada type-IV polyp with redness was discovered on the posterior wall of the upper part of the body of the stomach. Endoscopic findings were those of a typical HP. Endoscopic polypectomy was conducted.

### 3.3. Changes after 1994

Endoscopic examinations were carried out in 45 of the 95 HP patients after 1994. Cancerous foci were observed in 2 patients, in the monitored polyp in 1 patient and in other area in 1 patient.

Endoscopic examinations were carried out in 31 of the 84 FGP patients after 1994. Cancerous foci were observed in no monitored polyps or other areas in any patient.

### 3.4. Presence or Absence of *H. pylori*

Microscopic examinations revealed organisms suspected of being *H. pylori* in 56 of 95 HP patients (58.9%) (Figs. 4 and 5) and 27 of the 84 FGP patients (32.1%). Differences between the two groups were statistically significant ($p = 0.001$ by $\chi^2$-test) (Table III).

### 3.5. Degree of Inflammation

The degree of inflammation was determined by examining biopsy specimens taken from polyps.

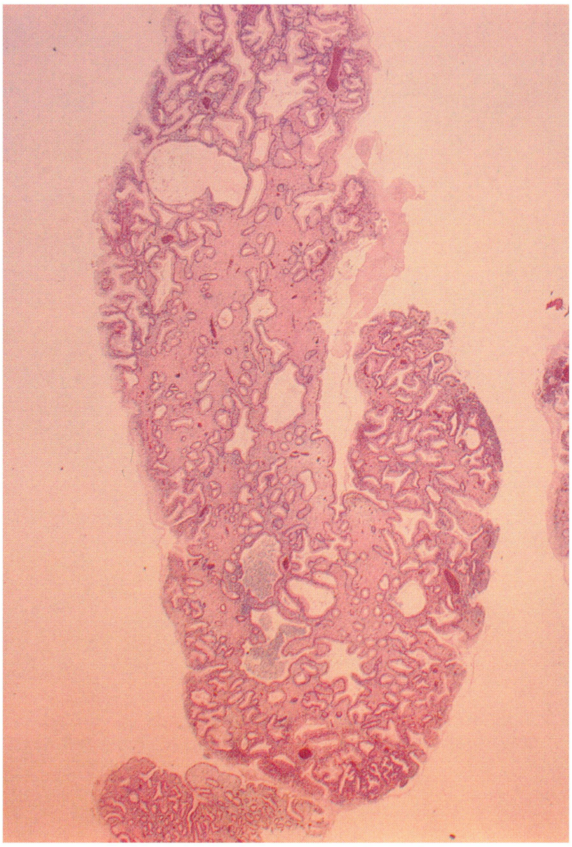

FIGURE 2 Case 1. Histological findings were consistent with those of an HP in most parts of the polyp.

Among the 95 HP patients, inflammation was absent in no patient, mild in 3, moderate in 32, and severe in 9 of 60 with a mean score of 2.6, while among the 84 FGP patients, it was absent in 0, mild in 67, moderate in 17, and severe in 0 with a mean score of 1.2. Differences between the two groups were significant ($p = 0.001$, $U$-test) (Table IV).

Findings outlined in Sections 3.4 and 3.5 showed that the *H. pylori* infection rate is higher and inflammatory cell infiltration more marked in HP patients than in FGP patients.

### 4. DISCUSSIONS

At present, malignant neoplasms is the No. 1 killer in Japan. Among malignant neoplasms, stomach

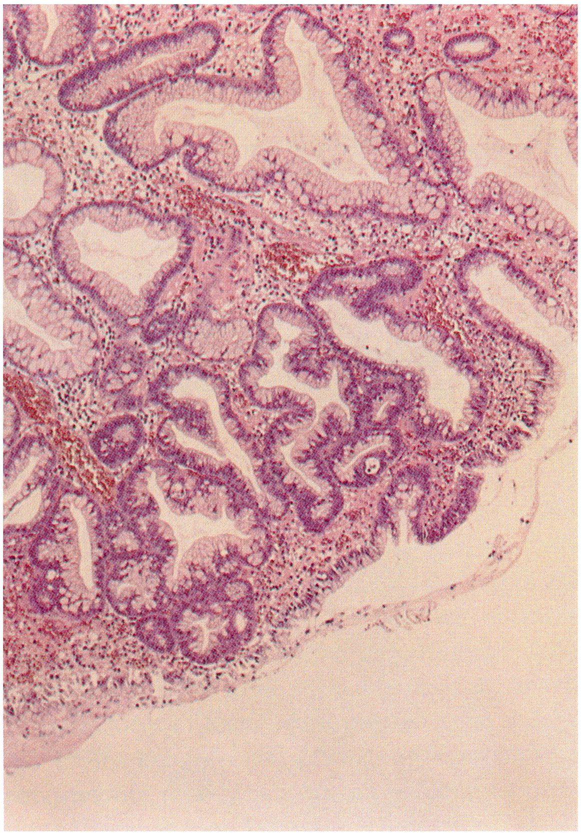

FIGURE 3 Case 1. A dysplastic focus was noted near the base of the polyp.

cancer is ranked No. 2 for men and No. 1 for women. About 50,000 persons die of stomach cancer each year. However, mortality due to stomach cancer has been declining since 1965 due to a number of factors such as early discovery and treatment through mass examination programs. Stomach cancer is discovered in only 0.1–0.2% of persons who participate in mass examinations, so they are not necessarily cost-effective in detecting stomach cancer patients. Due to the slow-down of economic growth in recent years, government subsidies for mass examinations have been reduced. For this reason, various attempts, such as introduction of the pepsinogen determination method, have been made in order to increase efficiency of mass examinations. It is important to decrease the number of endoscopic procedures in order to reduce not only the economic burden but stress on patients as well. In the present study, therefore, we evaluated if the number of endoscopic procedures can be reduced in the monitoring of benign elevated gastric epithelial lesions (gastric polyps), which can be relatively easily detected and are in fact detected in a large number of persons by X-rays in mass examinations. In our facility, gastric polyps are detected in 7.8% of persons by X-rays in mass examinations. This detection rate is similar to that in the literature. Usually, endoscopy is conducted for detailed examinations if lesions are discovered by X-rays. Even if lesions are found benign as a result of detailed examinations by endoscopy, it is often repeated the next year if lesions are detected again by X-rays. In other words, patients undergo endoscopy once a year once gastric polyps are discovered by X-rays. Whether this monitoring is necessary and useful is questionable because it is well established that FGPs are not cancerous and that it is extremely rare that cancer occurs in areas other than FGPs in the same stomach [2]. In contrast, however, HPs are reported to be cancerated in some patients [3–5]. Therefore, applying the same monitoring policy to HPs and FGPs may be cost-ineffective. In order to give an answer to this question, we evaluated morphological changes in HPs and FGPs and reviewed biopsy findings in patients with these lesions.

As reported by many authors [4,6], morphological changes were seen with both HPs and FGPs, but incidence was slightly higher with HPs.

Dysplastic or cancerous foci are often detected in excised HPs. In the present study, dysplastic foci were found in 30.8% of HP lesions, a very high rate probably partly due to the small number of lesions examined. In our previous study, dysplastic and cancerous foci were detected in 5% and 2.5%, respectively, of endoscopically excised HPs. In a similar study, Iijima [3] reported the presence of dysplastic and cancerous foci in 9.2% and 2.9%, respectively, of HPs and found that dysplastic lesions were tumorous, suggesting that dysplastic lesions may be transformed into cancer. These findings suggest that HPs for which polypectomy is indicated have cancerization potential. Histologically,

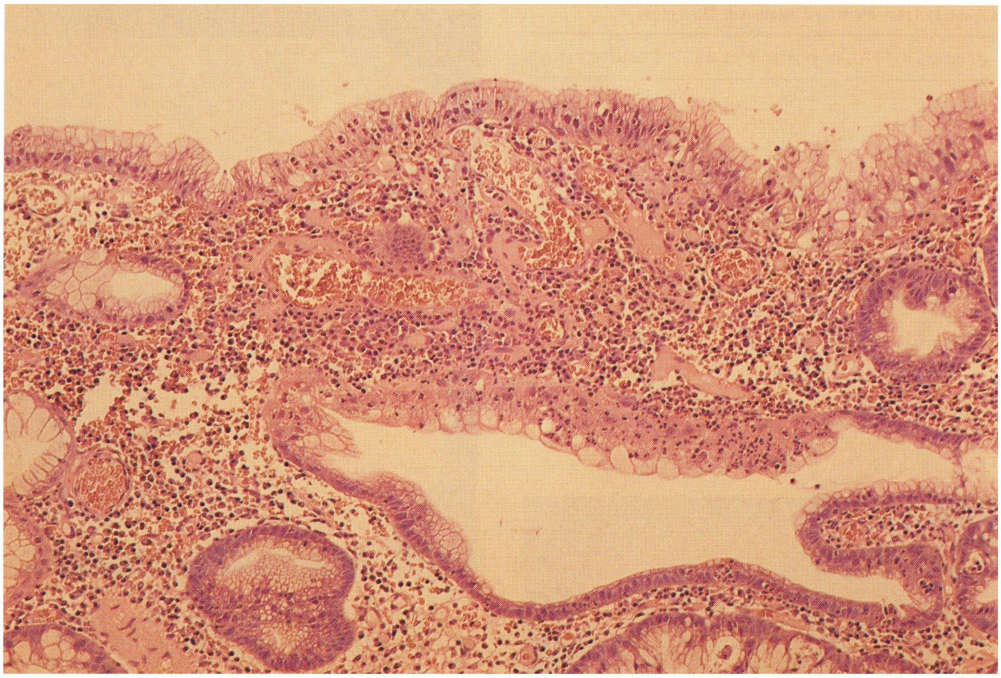

FIGURE 4 The foveolar epithelium shows hyperplasia. Marked infiltration of lymphocytes, plasmacytes, and neutrophils is seen in the interstitium. Proliferation of capillaries is also observed. It showed a typical histological finding of HP (HE ×200).

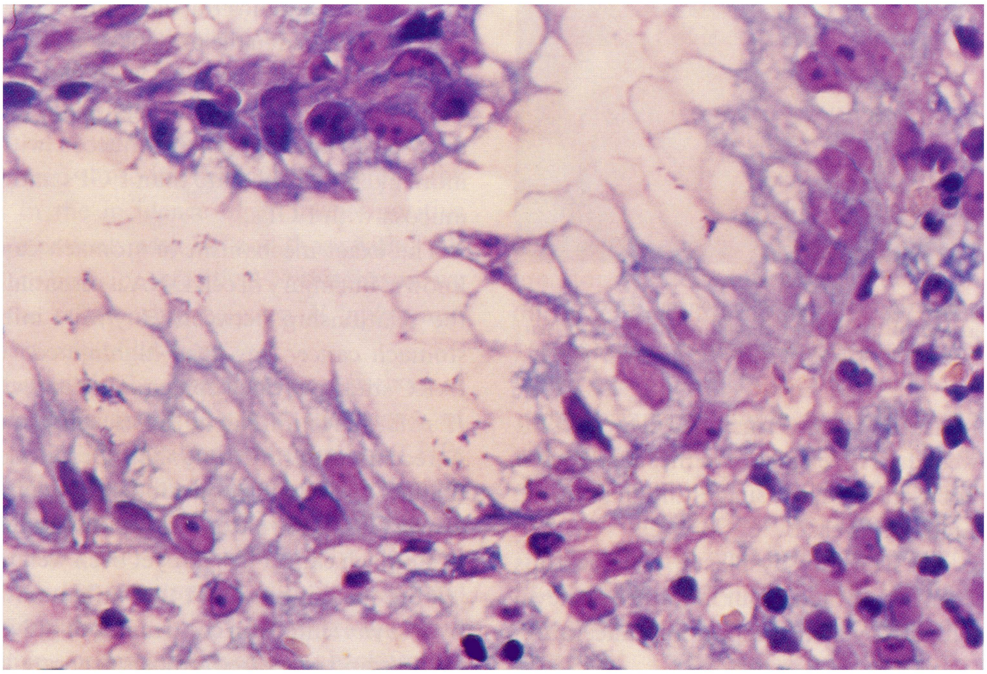

FIGURE 5 Mucus is found in the surface layer of this HP. *H. pylori* is found in the mucus and adhered to the surperficial epithelium (Giemsa ×400).

TABLE III Positive ratio of *H. pylori*

| | No. of cases | No. of positive cases | Positive ratio* |
|---|---|---|---|
| HP | 95 | 56 | 58.9 |
| FGP | 84 | 27 | 32.1 |

*$p = 0.001$ ($\chi^2$-test).

TABLE IV Degree of inflammation

| Score | None 0 | Mild 1 | Moderate 2 | Severe 3 | Average score* |
|---|---|---|---|---|---|
| HP ($n = 95$) | 0 | 3 | 32 | 60 | 2.6 |
| FGP ($n = 84$) | 0 | 67 | 17 | 0 | 1.2 |

*$p = 0.001$ ($U$-test).

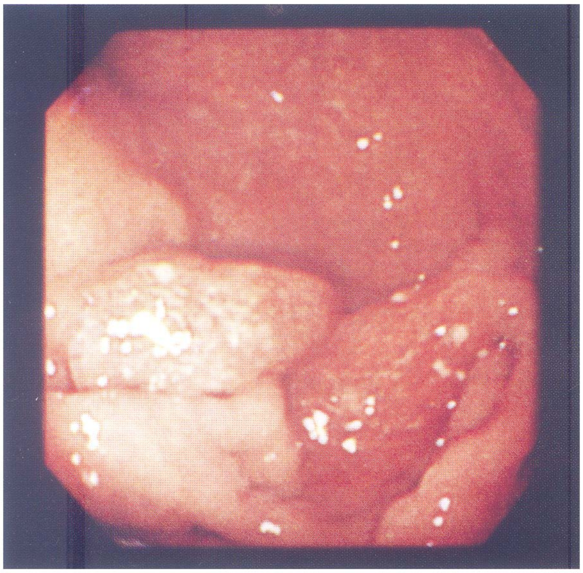

FIGURE 6 Case 2. A 60-year-old woman. Three polyps were discovered, 2 in the greater curvature in the lower part of the body of the stomach.

the lesion in case 2 (Figs. 6–9) included in the present study showed an HP element at the base and a cancerous focus at the apex, suggesting that an HP has cancerated in this case.

We have not seen any dysplastic or cancerous foci in FGPs in our patients. Our literature retrieval also failed to discover any report of canceration of FGPs.

Cancerous or tumorous lesions were detected in areas other than polyps in 3 HP patients (3.2%) but

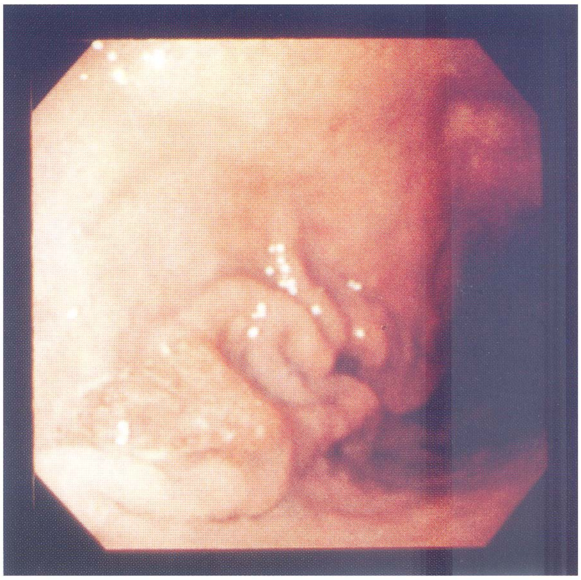

FIGURE 7 Case 2. The other polyps were discovered in the greater curvature in the middle of the body of the stomach. All polyps showed redness and were endoscopically diagnosed as HPs. All 3 polyps were endoscopically excised.

in no FGP patient. Furthermore, monitoring of patients included in the present study revealed onset of carcinoma or adenoma in 2 HP patients but in no FGP patient. These findings suggest that HPs themselves and the gastric mucosa with HPs are more likely to cancerate than FGPs and the gastric mucosa with FGPs.

The exact mechanism of stomach cancer is unknown, but injury of cell DNA is essential. Recently, the relationship between *H. pylori* infection and stomach cancer has been highlighted. The WHO IARC study provided epidemiological evidence that *H. pylori* infection can be a cause of stomach cancer. A recent study in Mongolian Gerbil also showed that *H. pylori* infection alone can cause stomach cancer [7,8]. The role of *H. pylori* in the development of stomach cancer has not yet been well understood [9], but there is no doubt that inflammation caused by *H. pylori* infection contributes to it. Neutrophilic elastase and other free radicals generated by inflammation caused by *H. pylori* infections react with ammonia produced by *H. pylori*'s urease, resulting

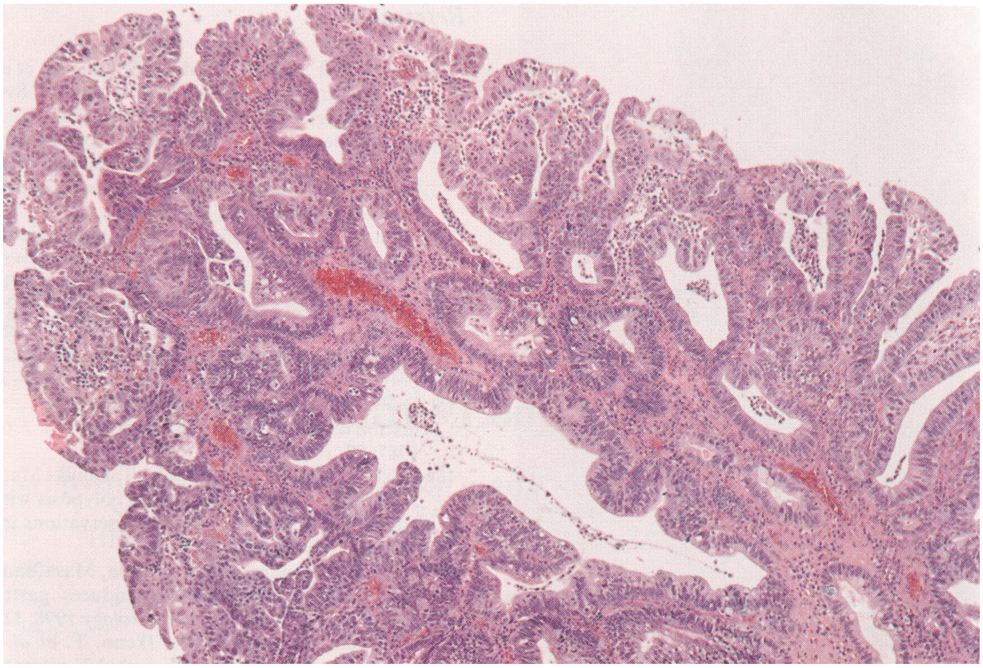

FIGURE 8 Case 2. Of the 2 polyps in the lower part of the body of the stomach, the one on the anterior wall showed well differentiated tubular adenocarcinoma in an area from the middle to the apex.

in the formation of monochloramine, which injure DNA of gastric epithelial cells [10,11].

Incidence of *H. pylori* infections is closely related to the ages of patients. In Japan, incidence of positive serum antibody is comparable in patients in their 50s and those in their 60s. Therefore, the differences in the incidence of *H. pylori* positive rate between patients with HP and those with FGP are thought to be lesion-specific. For this reason, we evaluated the relationship between *H. pylori* and inflammatory cell infiltration.

We determined the presence or absence of *H. pylori* in HPs and FGPs and the degree of inflammatory cell infiltration and found that HPs are more often infected by *H. pylori* and show more marked inflammation than FGPs. This finding seemed to offer a clue to the answer why HPs and the background gastric mucosa with it show great malignant potential.

Endoscopic examinations of the background gastric mucosa with HPs and FGPs show that atrophic changes are marked in the presence of HPs, while the gastric mucosa is almost normal or free from atrophic changes in the presence of FGPs [12]. This difference is also thought to be due to lower incidence of *H. pylori* infections and milder inflammation in the presence of FGPs.

All these findings suggest that yearly endoscopic monitoring is unnecessary in FGP patients, but our data is not sufficient to establish the appropriate monitoring interval for these patients. In addition, during the monitoring of one FGP patient in the past, we observed the disappearance of an FGP which was followed by the onset of an IIa-type early stomach cancer at another site [13]. A similar event was also reported in some patients. Therefore, FGPs do need monitoring. The question is how often and how. At this stage, we would recommend that X-rays be repeated once a year to compare films with those of the previous year and that endoscopy be repeated for detailed examinations if any change is observed.

HPs need to be endoscopically examined once a year because they have malignant potential as

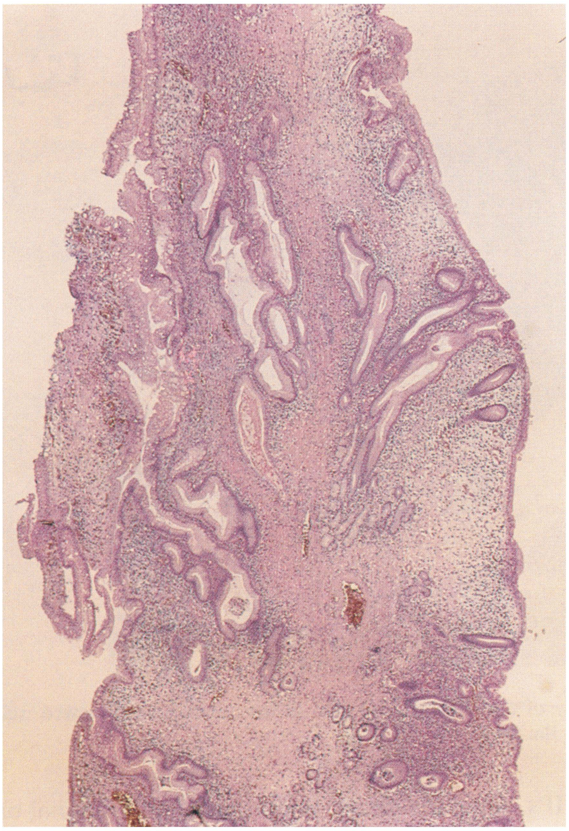

FIGURE 9 Case 2. Findings of an HP were obtained in the lower part of the polyp. The two other polyps were diagnosed as HP histologically.

shown in the present and other studies. In addition, biopsy needs to be conducted at the same time because it is difficult to accurately diagnose dysplastic or cancerous foci by endoscopy alone. In conclusion, yearly monitoring by endoscopy is necessary in the case of HPs, but X-rays may be omitted.

### References

[1] Dixon, M.F., Genta, R.M., Yardley, J.H. *et al.* Classification and grading of gastritis: The updated Sydney system. *Am. J. Surg. Pathol.* 1996; **20**: 1161–1181.

[2] Naomi Uemura, Kouji Sumii, Goro Kajiyama *et al.* The characteristic change of gastric mucosa in patients with fundic gland polyp and gastric cancer. *Gastroenterological Endoscopy* 1993; **35**: 2663–2671.

[3] Naoto Iijima. A study on the immunohistochemical and DNA ploidy patterns of gastric hyperplastic polyps removed by endoscopic polypectomy. *The Journal of Tokyo Medical University* 1991; **49**: 530–540.

[4] Janina Orlowska, Dorota Jarosz, Jacek Pachlewski *et al.* Malignant transformation of benign epithelial gastric polyps. *Am. J. Gastroenterol.* 1995; **90**: 2152–2159.

[5] Daibo, M., Itabashi, M. and Hirota, T. Malignant transformation of gastric hyperplastic polyps. *Am. J. Gastroenterol.* 1987; **82**: 1016–1025.

[6] Kazuoki Hizawa, Mitsuo Iida, Takayuki Matsumoto *et al.* Natural history of fundic gland polyposis without familial adenomatous coli: Follow-up observations in 31 patients. *Radiology* 1993; **189**: 429–432.

[7] Takeshi Watanabe, Mayumi Tada, Masafumi Nakao *et al.* *Helicobacter pylori* infection induces gastric cancer in Mongolian Gerbils. *Gastroenterology* 1998; **115**: 642–648.

[8] Sugiyama, A., Maruta, F., Ikeno, T. *et al. Helicobacter pylori* infection enhances N-methyl-N-nitrosourea induced stomach carcinogenesis in the Mongolian Gerbil. *Cancer Research* 1998; **58**: 2067–2069.

[9] Shoko Midorikawa, Minoru Kawaguchi, Yutaka Handa *et al.* Correlation between gastric carcinogenesis and *Helicobacter pylori* infection from the viewpoint of histopathology, cell proliferative activity, and gene mutation. *The Journal of Tokyo Medical University* 1998; **56**: 731–740.

[10] Iishi, H. *et al.* Enhancement by monochloramine of the development of gastric cancers in rats: A possible mechanism of *Helicobacter pylori* associated gastric carcinogenesis. *J. Gastroenterology* 1997; **32**: 435–441.

[11] Tomb, J.F. *et al.* The complete genome sequence of the gastric pathogen *Helicobacter pylori*. *Nature* 1997; **388**: 539–547.

[12] Ken Haruma, Kouji Sumii, Akihiko Morikawa *et al.* Gastric mucosa in patients with fundic hyperplastic polyps. *Japanese Journal of Gastroenterology* 1989; **86**: 851–857.

[13] Tetsuya Sanji, Minoru Kawaguchi, Toshihiko Saito *et al.* A case of early gastric cancer following natural disappearance of fundic gland polyps. *Progress of Digestive Endoscopy* 1997; **50**: 270–271.

